# The paradigm of eave tubes: scaling up house improvement and optimizing insecticide delivery against disease-transmitting mosquitoes

**DOI:** 10.1186/s12936-017-1859-z

**Published:** 2017-05-19

**Authors:** Fredros Okumu

**Affiliations:** 1 Environmental Health and Ecological Sciences Department, Ifakara Health Institute, Ifakara, Tanzania; 2 School of Public Health, University of the Witwatersrand, Parktown, Republic of South Africa; 3 Institute of Biodiversity, Animal Health and Comparative Medicine, University of Glasgow, Glasgow, UK

## Abstract

Control of mosquito-borne diseases is greatly compromised by spread of insecticide resistance, high implementation costs and sub-optimal compliance among users. Improved housing has potential to reduce malaria transmission nearly as much as long-lasting insecticide-treated nets (LLINs), while also preventing other arthropod-borne diseases and improving overall well-being. Yet, it is hardly promoted as mainstream intervention, partly because of high costs, minimal communal benefits to people in non-improved houses, and low scalability. By exploiting biological observations of mosquito behaviours around dwellings, scientists have developed a new approach that integrates effective vector control into housing developments. The technique involves blocking eave spaces in local houses, leaving a few cylindrical holes into which plastic tubes with insecticide-laden electrostatic nettings are inserted. Where houses already have blocked eaves, these cylindrical holes are drilled and the tubes inserted. The eave tube technology, as it is called, is an innovative new approach for implementing housing improvements, by creating a new scalable product that can be integrated in houses during or after construction. It takes away insecticides from proximity of users, and instead puts them where mosquitoes are most likely to enter houses, thereby reducing insecticidal exposure among household occupants, while maximizing exposure of mosquitoes. This way, lower quantities of insecticides are used, better house ventilation achieved, intervention costs reduced, and mass communal benefits achieved even were vectors are resistant to similar insecticides when delivered conventionally. There are however still some critical pieces missing, notably epidemiological, social and economic evidence that the above assertions are true and sustainable. Besides, there also some technical limitations to be considered, namely: (1) need for extensive house modifications before eave tubes are inserted, (2) ineligibility of poorest and highest-risk households living in housing structures not amenable to eave tubes, and (3) poor synergies when eave tubes are combined with LLINs or IRS in same households. Overall, this paradigm significantly improves delivery of insecticides against disease-transmitting mosquitoes, and provides opportunities for scaling-up the long-neglected concept of house improvement as a malaria intervention.

## Background

Malaria control has come a long way since the last attempts to achieve global eradication [1] and the lull that followed abolition of vertical disease control programmes and adoption of primary health care concept in the 1970s [2]. In the period after the famous Abuja declaration by African presidents in 2000 [3], significant investments have been made on malaria control, resulting in substantial gains from more than one million deaths annually to just over 400,000 malaria-related deaths in 2015 [4]. Today, most endemic countries use insecticide-treated nets (ITNs) and indoor residual spraying (IRS), both of which are estimated to have contributed to ~78% of all malaria cases averted since 2000 [5]. Other interventions commonly stipulated in national malaria control policies include larval source management especially in urban locations [6], prompt diagnosis and improved treatment options as well as malaria prevention in pregnancy and health education. Despite all the successes so far, several challenges still abound. Meeting the current targets as outlined in the Global Technical Strategy [7] will, therefore, require major improvements on how the burden of disease is controlled and assessed.

Vector control has clearly been the mainstay of malaria prevention but sustaining these gains will require complementary approaches not constrained by factors, such as low user compliance, insecticide resistance, high costs, or behavioural adaptations of vector populations [8]. In pursuit of the sustainable development goals [9], an important additional characteristic for such new tools is that they should be integrated in people’s way of life, such that associated gains may exceed disease prevention to also include improvements in overall well-being.

Evidence that house improvement can prevent malaria transmission has been plentiful for hundreds of years; people living in screened houses experience fewer mosquito bites that those in unscreened houses. In a recent review, Tusting and colleagues demonstrated, through an extensive review of multiple Health and Demographic Surveillance (HDSS) data and also Malaria Indicator Survey (MIS) datasets that improved housing (modern houses) offer nearly as much epidemiological benefits against malaria as ITNs [10]. Yet, the level of this evidence is still considered weak and to date, and there are no associated cost-effectiveness data comparing it with established approaches like LLINs and IRS. The World Health Organization (WHO) has not provided any unequivocal recommendation for countries to consider house improvement and many countries do not have clear national policies for integrating house improvement in disease prevention.

While there are concerns that outdoor-biting mosquitoes are becoming an increasingly important component of residual malaria transmission across Africa, recent evidence suggests that most outdoor transmission is mediated by mosquitoes that have previously been indoors [11]. Besides, large-scale entomological surveys in rural Tanzania have demonstrated that even in areas where outdoor biting constitutes 70% of all mosquito bites on humans, most *Plasmodium*-infected mosquitoes are still found indoors (Okumu et al. unpublished data). Improved house-based interventions therefore remain relevant for both malaria control and elimination.

## Socio-economic transition in Africa and its impact on housing designs

While ITNs and IRS have undoubtedly become the most scaled-up and also most effective malaria prevention tools [5], the underlying market forces and funding approaches are predominantly external, suggesting that continued sustainability may not be guaranteed. Failure to establish local production or vibrant private sector support in most user countries could exacerbate this problem, in event of reduced external support. On the contrary, an interesting observation across Africa is that even without any direct overseas financial support, the quality of individual human dwellings across villages and towns is gradually improving. For example, proportions of African households with iron or tiled roofs, and brick or concrete walls, as opposed to other building materials such as thatch, mud and reeds, have on a consistent upward trend. In some countries, these have been promoted through government-backed programmes, such as regulatory enablement for private homes and building materials, as well as government housing programmes [12, 13]. However, the majority of the improved houses in Africa are paid for directly through individual household incomes; driven primarily by improved living standards and the innate desire for better quality housing.

In 2011, the African Development Bank estimated that the African middle class had already reached 313 million people, just above 34% of the total population at the time [14]. This was driven by strong economic growth and a move towards stable salaried job cultures as opposed to traditional agricultural activities [14]. The growth has resulted in significant increase of household expenditures as well as higher investments in improved housing and housing accessories by families. 8 years ago, the market size for window screens and ceilings used for house improvements against mosquito bites in Dar es Salaam, Tanzania, was already significantly higher than market size for LLINs [15]; and more than 80% of households were already using these accessories in one form or another. Based on these estimates, Ogoma et al. concluded that such products would be highly acceptable and taken up in many tropical cities and towns [15]. Together with floor tiles, wall paint and fencing, these voluntary house improvement investments already exceed average contributions that individual households pay for many other vector products in Africa.

These improvements indicate an innate desire by people to have better housing and offer multiple opportunities to integrate innovative disease control measures in these homes. Besides, they demonstrate the presence of a vibrant, locally driven market for housing improvement, which could be exploited to support scale-up of integrated vector control even in relatively low-income communities. Unlike stand-alone vector control products, such as LLINs, effective new tools integrated into buildings would require minimum user compliance, yet offer longer-lasting protection for all household members, even if the initial financial outlays are marginally higher than costs of IRS, LLINs or other vector control products. Hence effective models for promotion and subsidization should be developed to promote home products that prevent entry of disease-transmitting and nuisance biting mosquitoes [15].

## The eave tubes

**Fig. 1:**
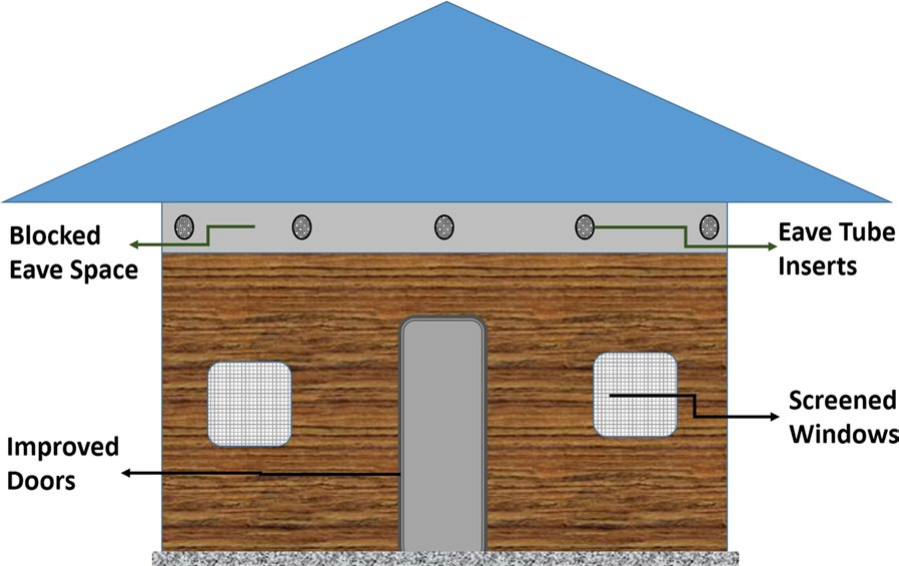
A simplified representation of the eave tubes approach (figure not drawn to scale). According to published specifications, the eave tubes approach, includes much more than just the inserts. Instead, the eave spaces are also blocked so that the only remaining spaces are those for inserting the tubes. In addition, any windows available are also screened, wall cracks sealed and doors reinforced to seal any openings. As a result, though the technology is referred to as simply as “Eave Tubes”, it actually a combination of traditional house improvement and the actual eave tube inserts

In a series of articles published between August 2016 and May 2017, a team of scientists, funded primarily through a European Union Seventh Framework Programme for Research (EU’s FP-7), have described an innovative new approach for integrating vector control, as a component of house improvement against mosquito-borne diseases [16–18]. The papers explain development and evaluation of technology and its various components, but also highlight its potential as a high impact intervention against malaria and other mosquito-borne infections in Africa. Though known simply as “Eave Tubes”, which is an obvious but clever misnomer, the technique involves blocking eave spaces (i.e. spaces between roofs and the top of walls) in local houses, leaving multiple cylindrical holes (~1 m apart), in which PVC pipes with insecticide-laden nettings are inserted (Fig. 1). In cases where houses are already fully build without these eave spaces, the cylindrical holes can be drilled along the upper surfaces of these walls (usually at ~1.7 to 2 m heights above ground, just under the roofs), then the PVC pipes, which are the actual eave tubes are inserted. In addition to the modifications around eave spaces, any windows available are also screened, wall cracks sealed and doors reinforced to seal any openings [16].

The biological relevance of this approach is obvious. Host-seeking mosquitoes typically fly upwind towards the source of host odours. Channeling the flow of human odours from dwellings can be used as means to lure and kill the mosquitoes at the eaves or windows. *Anopheles* mosquitoes, which transmit malaria and lymphatic filariasis predominantly enter human dwellings through eaves, and exit either through the same openings or via open windows and doors [19, 20]. Other species such as *Culex* and *Mansonia* mosquitoes also use these spaces, though in some situations they have been observed to prefer entering via open windows and doors and exiting via eave spaces, windows and doors [20]. Where the eave tubes are the only available openings, they could form a highly targeted option for controlling human-biting mosquitoes. Lindsay’s group, working mostly in the Gambia, have previously elucidated in great detail, the challenges and possible solutions related to open eave spaces [19, 21, 22]; and also demonstrated epidemiological evidence of house screening [22]. The relevance of eave spaces for vector control can also be inferred from previous work involving direct video graphic observations of malaria vectors entering houses [23]. Upon arrival at the eave area, mosquitoes greatly reduce speeds and assume highly convoluted flight paths, spending several seconds in the near-proximity of the eaves before eventually entering the huts [23].

Insecticidal eave tubes are thus ideal for controlling mosquitoes that bite people and/or rest indoors, which are also the most epidemiologically relevant populations of these vectors. Other than killing the potentially-infectious mosquitoes, the technology has several additional benefits to consumers. For example, in cases where houses do not already have enough openings, the eave tube inserts provide additional ventilation, potentially improving airflow and air quality inside the houses, depending on the overall surface area of the eave tubes relative to total volume of the house.

## Design principles, target product profile and development approaches used for the eave tubes technology

In their introductory article, Knols et al. provide the background of the eave tubes technology, highlighting its origins, conceptualization and relevance [16]. From the very onset, there are two critical items that will likely remain relevant for future vector control products. First, is the approach used by the team, tackling the issue primarily as a problem to be solved, and not purely as an academic exercise. While paying due attention to methodical identification and pursuit of specific academic questions and hypotheses, the eave tubes team began by observing a wide range of house designs in rural African communities. They also observed house improvement trends and preferences of community members. This was followed by a non-conventional brain-storming session on how best to address the problems they had observed first-hand, using the simplest options possible. In a blog post written shortly after this event, in February 2014, Bart Knols, who led the team spoke about how they sat under a large mango tree overlooking the village, and how they used sticks to illustrate the ideas on the sand, instead of PowerPoint slides, projectors and computers [24]. Here it is also important to note the unique composition of the team, which included not only mosquito biologists, but even more critically, hands-on engineers, entrepreneurs and material scientists, some with previous experience developing products. The team members were drawn from five institutions in five countries and three continents (Pennsylvania State University, USA; CTF2000, Belgium; Biogents, Germany; Ifakara Health Institute, Tanzania; and In2Care BV, Netherlands), and had an advisory committee extending this to four continents.

The second important item was the basic minimum target product profile (TPP), upon which development of eave tubes was anchored [16]. This TPP was establish a priori, and included eight essential criteria for effectiveness, but had no additional desirable characteristics indicated. In summary, the developers of eave tubes decided that a successful product would be that which: (1) minimizes contact between household dwellers and insecticides used for vector control; (2) enables application of novel chemicals including biological control agents or combinations of different classes of agents, (3) significantly reduces the amount of chemicals required, and potential exposure of environment and non-target organisms, (4) can be used together with existing interventions without compromising overall efficacy, (5) minimizes need for user compliance or user involvement, (6) can be integrated in day-to-day way of life, without excessive additional inputs, (7) enables income generation, mass production, scalability and ease of use, and (8) eliminates need for external energy sources or supplies, e.g. electricity and mosquito attractants. Though originally stipulated as part of the TPP for eave tubes, these criteria will most likely transcend new vector control tools being developed today, including those specifically meant for malaria elimination. An additional property that may be included in this list, perhaps as a desirable component of the TPP, is the need to provide mass communal protection beyond just the users, and the need to improve overall wellbeing and living standards, rather than simply being a vector control product. This way, vector control products can align more effectively to the SDG goals including the third goal, to promote well-being and ensure healthy lives at all ages, as well as goals 10 and 11 [25].

Having set the ball rolling, the eave tubes technology was submitted for patent protection, alongside other five vector control products, long before any studies or further development was done, another difference in how this group approached vector control [26]. A key lesson here is that early assessment of both social and financial impact of any new tool should be conducted soon after conception and where necessary, the essential intellectual property protections sought, not with a view of maximizing the financial benefits, but to maximize unrestricted access to the neediest communities at reasonable cost.

## Effects of eave tubes combined with eave blocking and window screening on mosquito densities and survival

Standard house improvement approaches, such as blocking eave spaces already provide substantive household-level protection. However, re-opening parts of these eave spaces, and inserting PVC tubes with insecticidal screens, effectively turns houses into vector killing stations. Mosquitoes are lured to these specific points, and killed en masse, thus achieving communal protection, even for residents in unimproved houses.

**Fig. 2:**
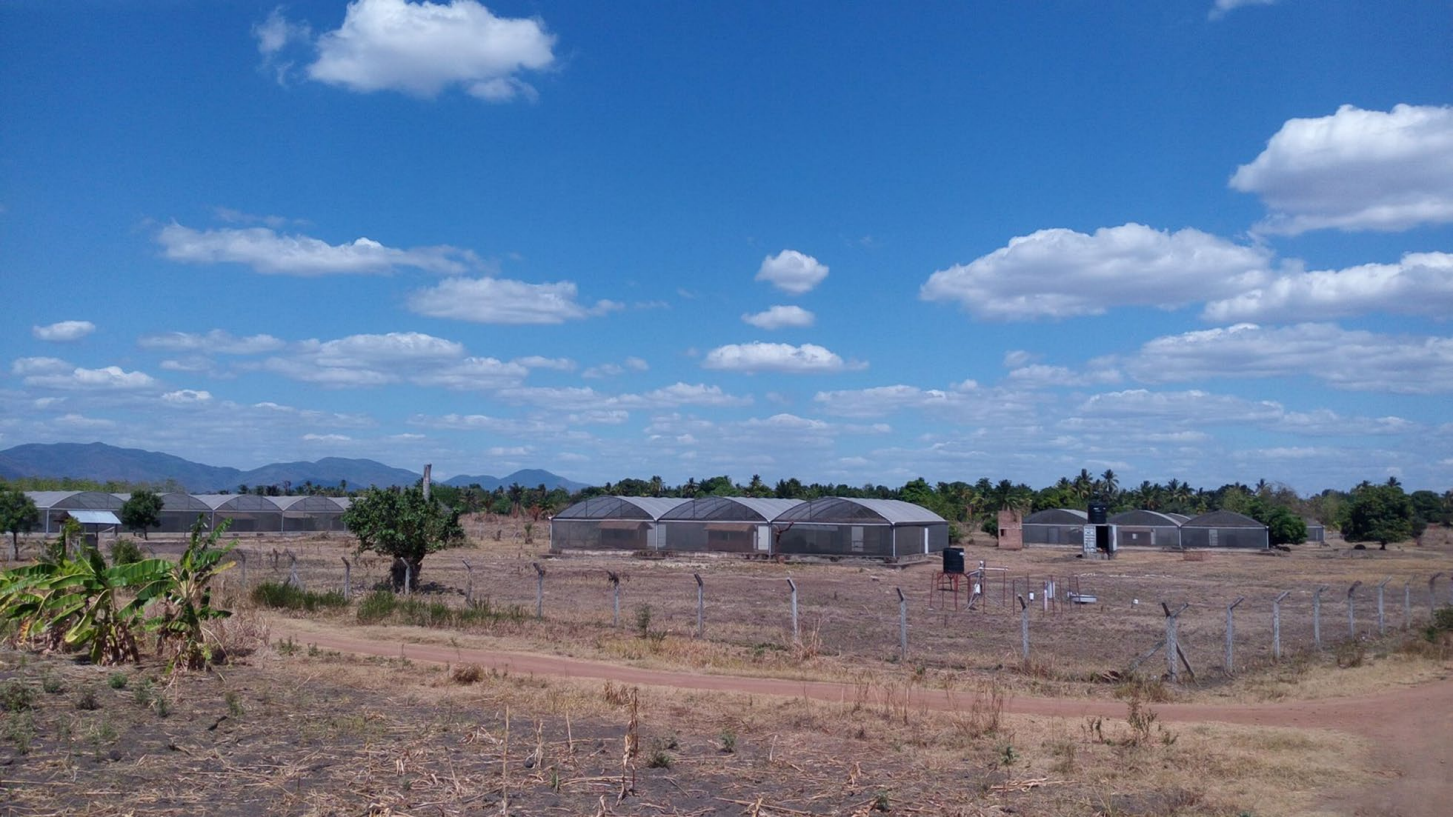
The Mosquito City: a pictorial Illustration of the semi-field facilities at Ifakara Health Institute, in Tanzania, where new vector control products are evaluated before they are taken for full-field studies. The eave tube technology was initially tested here, against self-sustaining colonies of the malaria vector, *Anopheles arabiensis*, in an ecosystem mimicking rural African villages. The model village had houses built to mimic both modern and traditional designs, human volunteer sleepers working at night, cattle, model rice fields, multiple forms of vegetation, as well as insecticide treated bed nets, the primary malaria intervention already widely used in local villages

In their second article in the series [17], the team describes initial tests, and provide empirical evidence on basic functionality of the eave tubes. These initial studies were conducted inside large semi-field enclosures at Ifakara Health Institute, in what is the largest such facility ever built for mosquito research (Fig. 2). Experimental huts mimicking local houses in Tanzania were constructed inside different chambers of the semi-field facility, and used to iteratively evaluate and optimize the technology. Various basic aspects were examined, including: (a) effects of varying diameters of the eave tubes, (b) optimal height above ground and angle of the tubes and (c) efficacy of different insecticides. The team also compared efficacy of the eave tubes relative to LLINs inside experimental huts in the semi-field. To assess potential of this technology in complex ecosystems, they created a model village inside the semi-field cages, with self-sustaining colonies of the malaria vector, *Anopheles arabiensis*, surviving off sugar and cattle as the main blood-meal source, but with human volunteer sleepers as well. Though not exactly similar to natural ecosystems, this setting was the nearest possible to what one finds in rural African villages, with multiple vertebrate hosts. The team conducted tests where LLINs, followed by combinations of LLINs and eave tubes plus window screens were introduced, and vector densities monitored indoors, outdoors and in aquatic habitats, before, during and after each intervention arm [17].

An interesting finding from these early studies was that even by simply closing the eave spaces and inserting empty eave tubes without any netting, mosquito house entry could be reduced by approximately three quarters. An obvious interpretation here is that with regard to household-level protection, most of the overall benefits of the eave tubes approach are accrued from the physical blocking of the eave spaces, and screening of the windows. The main benefit of adding the insecticidal inserts is, therefore, the extra-communal benefits associated with mass killing of mosquitoes. Indeed, the investigators also demonstrated equivalent or greater reduction of vector densities indoors and in peri-domestic spaces when eave tubes were used relative to when LLINs were used, demonstrating potential of the technology for both household level and communal protection [17]. The eave tubes used in these tests were fitted with insecticidal nets cut from commercially available LLINs, or nets treated with bendiocarb (a carbamate insecticide), the latter providing marginally higher protection than LLINs.

The more striking findings in these initial tests were from the semi-field observations in the model village, where monitoring was done longitudinally over one year [17]. Introducing LLINs in the model village with a 4-month old *An. arabiensis* colony reduced densities of the mosquito larvae by 58%, and indoor adult densities by 85%. The researchers then waited a further 2 months, after which they introduced insecticide-treated eave tubes together with window screens in 4 of the 6 houses. At this point, larval densities were further reduced by 84% and indoor biting was completely eliminated. The mosquito densities remained suppressed until after withdrawal of the eave tubes and window screens, when they began rising steadily, reaching the LLIN-only levels after a further 3 months. Thus, in a period of just 1 year, the team had completed full development and basic evaluations of the core of this technology; and also identified key gaps to be pursued in the years ahead.

One limiting aspect highlighted here was that while LLINs can be used in all houses regardless of design and material, eave tubes are not easily fitted onto mud-walled houses. In these semi-field tests, the authors inserted the tubes only on brick-walled huts (modern houses), but not the mud-walled (traditional houses). Though African houses are improving gradually and proportions of with iron roofs and brick or concrete walls increasing, there are still millions living in houses with thatched roofs and/or mud walls. The implication here is that eave tubes may not be universally applicable.

A specific limitation relevant to these early semi-field observations was the lack of data on whether the self-sustaining mosquito colony had reached ecological stability, given that tests began only 4 months after the field larvae were introduced in the model village. Since the vector population was observed to be rising when eave tubes were withdrawn, it is reasonable to assume that presence of the tubes had been associated with the observed decline in the middle of the study. However, with this data alone, it cannot be concluded that the vector population was stable, or that the trends observed before, during and after the introduction of the intervention were wholly unnatural. The authors did not provide any descriptions of population dynamics, species composition (if there was more than one species in the system), filial generations, or natural temporal density variations across seasons. There was also no control data, from semi-field chambers with no interventions. Whereas a before-and-after approach, as used here, provides some indications of what might happen when interventions are introduced, dynamic and complex biological systems like vector populations, often have their own natural peaks and troughs, best monitored by a naïve but similar parallel population. Given the semi-field system was devoid of natural precipitation, vector dynamics in the model village could have been unaligned to natural rainfall seasons, adding a secondary layer of complexity. Addition of a control chamber with similar vector populations, tracked over the same period of time, would have provided an opportunity to rule out any alternative interpretations of the findings.

## Use of electrostatic coating in the eave tubes ensures high efficacy of insecticides despite resistance to conventional applications

The eave tube technology includes an innovative insecticide delivery system that maximizes effects of even small quantities of the active ingredients. By applying electrostatic coatings on the eave tube netting, instead of regular screens, mosquitoes that make contact with the netting are effectively “doused” with the active ingredients, ensuring high mortality even where susceptibility to the same insecticides, as measured by standard WHO assays, is already compromised. In an earlier publication in 2015, the group described in detail how this electrostatic coating works on the nets [27]. In summary, the coating binds to particles of any insecticide through polarity, and can retain the charge despite multiple washes [27]. Versions of this technology already existed for various applications including in air cleaners and filters [28], pollen binders on window screens for prevention allergic exposures [29], and even hair dryers [30], but it had also been used to deliver aerial insecticides [31] and mating disruption pheromones [32] to control agricultural pests.

Between 2014 and 2015, the potential of this technology for public health pesticide delivery was demonstrated by the eave tubes team in a series of smart experiments using laboratory and field-collected strains of susceptible and insecticide resistant *Anopheles, Culex* and *Aedes* mosquitoes from multiple African countries [27]. By exposing resistant mosquitoes to reduced doses, and for shorter periods than specified in WHO standards [33], they observed that electrostatic coatings significantly enhanced insecticide efficacy, effectively breaking insecticide resistance in mosquitoes, as currently defined in WHO standard susceptibility assays. This observation was true even with insecticide doses that were 15-fold lower than standard public health applications, and exposure times as low as 5 s compared to the 60 min in standard WHO assays [27].

While there are still various questions on how best to use the technology, the key expectation is that such coatings will be effective even where physiological insecticide resistance, particularly pyrethroids resistance, is widespread, and were the chemical classes used are same as those already being used for LLINs. While the global health community is racing to find new active ingredients for vector control, available options remain extremely limited. There are currently only 12 insecticides, in four different classes approved for IRS, six chemicals, all of which are pyrethroids approved for ITNs, five insecticides in two classes approved for space spraying, and ten insecticides in six classes approved for larviciding [34]. The urgency of innovative ways to use all available options is therefore obvious, and utility of electrostatic coatings clearly transcends the eave tubes technology. It presents many new opportunities to improve vector control and halt the spread of insecticide resistance.

Longer-term epidemiological studies will be required to ascertain these impacts in larger areas and to determine whether the enhanced insecticide delivery using such electrostatic coatings may have negative effects, e.g. by increasing insecticide pressure on vector populations, and consequently raising the intensity of resistance. Fortunately, the electrostatic coatings enable treatment of products like the eave tube inserts with different classes of insecticides, including non-pyrethroids such as carbamates, organophosphates, insect growth regulators or even biological control agents such as entomopathogenic-fungi, so the risk of spread of resistance can be minimized. In the initial semi-field tests, the coatings were used to deliver a carbamate, bendiocarb, rather than pyrethroids [17]. The team had earlier also used these coatings to dispense carbamates, pyrethroids and organophosphates and the mosquito-killing fungi, *Beauveria bassiana*, and suggested that the coatings could also deliver mixtures or mosaics or insecticide classes.

There are multiple other complementary benefits of using the electrostatic coatings on eave tubes. For example, these coatings limit any negative adsorptive effects of binding agents commonly used for insecticide formulation and also substrates used on sprayed surfaces, thereby effectively increasing overall bioavailability of the active ingredients. The eave tubes team demonstrated these benefits using lower doses and also lower exposure times [27]. Second, the electrostatic charge on the eave tube inserts can remain effective for months, making it desirable for both long-lasting insecticide delivery and resistance management. The increased bioavailability may also imply that the need for expensive carrier formulations or regulated heating as required for impregnation of nets with pyrethroids, may also be discounted.

## Focusing on eaves enables safer and more effective use of public health insecticides in households

Current indoor insecticidal interventions, IRS and LLINs, require relatively large treatment surfaces and high insecticide doses, and are subject to daily human handling or physical. A typical IRS dose is 0.02–0.03 mg/m^2^ for pyrethroids and 1–2 gm^2^ for carbamates, organophosphates and organochlorides, while ITN doses are 20–50 mg/m^2^ for most pyrethroids and 200–500 mg/m^2^ for permethrin and etofenprox [34]. In past years, there had been some suggestions to use insecticidal durable wall linings, some versions of which had as much as 172 mg/m^2^ of deltamethrin and were developed to cover the entire indoor walling of houses. Though excess bioavailability of the active ingredients in LLINs can be attenuated by use of binding agents and coating resins or by impregnation of the chemical into the netting fiber, most IRS applications, except some micro-encapsulated formulations, leave the insecticide directly exposed on the house surface, where they function purely as contact or airborne formulations. Also, insecticides in IRS decay rapidly, in most cases becoming non-effective after 3–6 months thereby requiring multiple treatments annually [34, 35]. Even with the new long-lasting formulations of pirimiphos-methyl, i.e. Actellic^®^ 300 CS [36], annual re-treatments are still necessary. Other than the challenges associated with using such large amounts of insecticides inside people’s homes, occupational hazards associated with the treatment and handling processes may also multiply.

Technologies that focus on the eave spaces enable us to keep insecticidal surfaces away from users, particularly children. The chemical active ingredients are put, in the eave tube inserts, in places where they are likely to have maximum impact, yet as far as possible from reach of occupants. The approach also allows pre-fabrication and standardization of insecticide applications, so users do not have to apply the insecticides themselves, and field operators can avoid steps for insecticide formulation and associated disposal as well as protection for handlers.

**Fig. 3:**
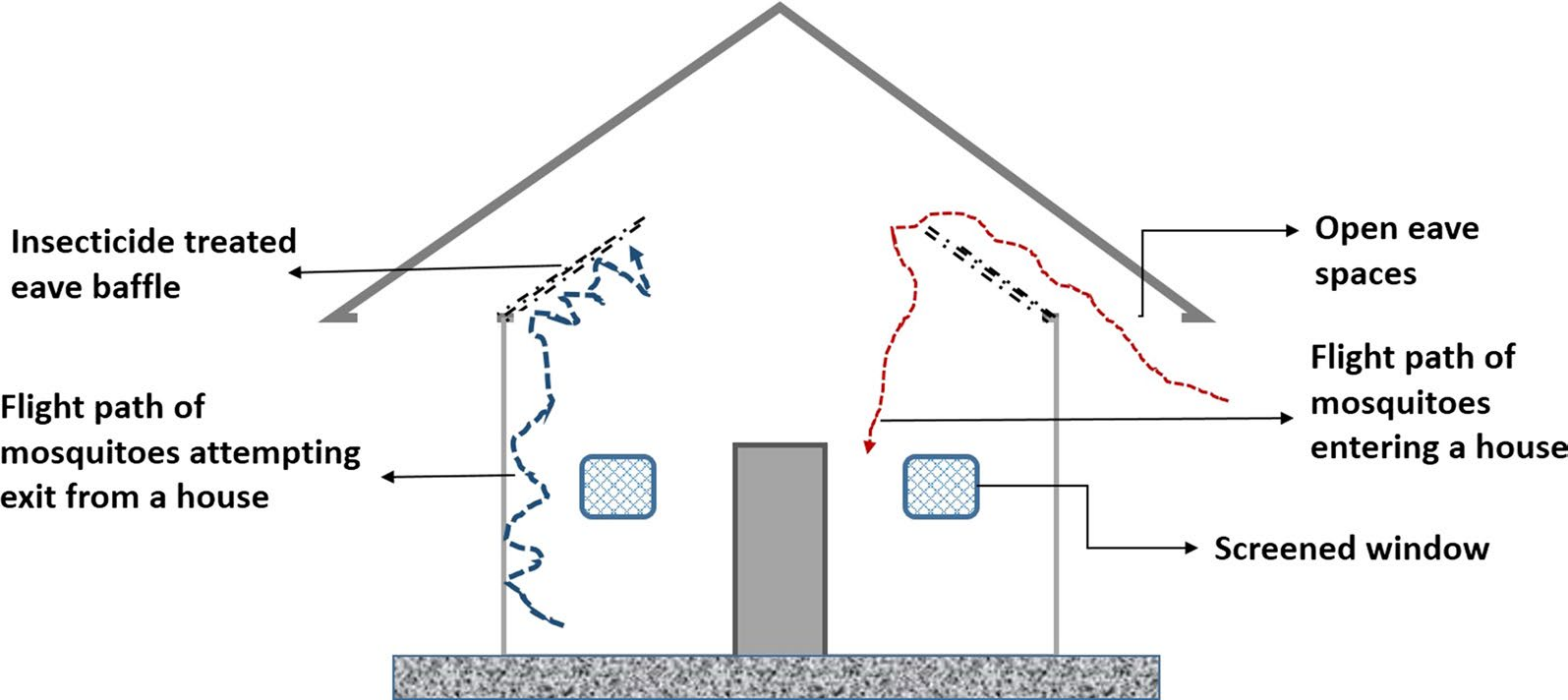
A simplified representation of the eave baffles approach, showing both the eave baffles, open eave spaces and screened windows (figure not drawn to scale). Unlike in the eave tubes approach, the eave baffles allow mosquitoes to fly in but restrict exit, thereby maximizing insecticidal contact as these mosquitoes attempt to exit. The windows are screened to maximize number of mosquitoes attempting eave exit, and also to increase lethal surface areas, as these window screened are also insecticide-treated. This allows greater synergism when the approach is combined with LLINs

The need to deliver insecticides safely and in lower quantities remains an important challenge, and has been considered in various alternative ways, arguably applicable across all house types. Most recently, a related technology with similar advantages and more has been developed [37], also at Ifakara health Institute, in Tanzania, where the first eave tube trials were conducted (Fig. 3). Here, Killeen et al. evaluated combinations of insecticide-treated window screens and eave baffles (WSEBs) for control of malaria vectors in rural Tanzania [37]. Eave baffles are pieces of slanting netting that rise at approximately 45° angle inwardly from the top of the wall to the roof allowing a 2–3 cm gap for host-seeking mosquitoes to enter. This is an adaptation of an old technology originally used in experimental hut studies where researchers noticed that some mosquitoes were escaping from the huts during field experiments [38]. They introduced inward and upward slanting barriers on top of the walls of huts (eave baffles), that directed mosquito movement to allow entry but prevent exit. The barriers were originally truncated cones made of plastic mosquito gauze or wire mesh that slanted towards the apex of the roof at approximately 2 cm away from but parallel to the roofing [39].

In the recent study by Killen et al. [37], it was demonstrated that window screens and the eave baffles treated with effective chemicals can offer equivalent protection as IRS with same chemicals, but with five times less insecticide annually. Similar to the eave tubes, WSEBs are vastly scalable, and if a binding agent is added to retain the insecticides for long, then the re-treatment frequency can also be reduced multi-fold. When the WSEBs were evaluated against standard IRS with same chemicals in Tanzania, the WSEBs killed similar proportions of pyrethroid-resistant *Anopheles funestus* (typically indoor-biting and indoor-resting) and higher proportions of pyrethroid *An. arabiensis* (typically opportunistic and readily feeding outdoors). Also as with the eave tubes, using non-pyrethroids, in this case the organophosphate, pirimiphos methyl, on the WSEBs, significantly magnified the benefits, indicating here too that both improved delivery and careful selection of active ingredients are key. More importantly, WSEBs can enable effective and scalable deployment of lower unit quantities of insecticides or combinations of insecticides against both susceptible and physiologically resistant mosquitoes [37]. Considering the fact that eave baffles allow mosquito house entry but restrict their exit, and since even outdoor biting mosquitoes are known to visit houses at least once in their life cycle [11], the WSEBs could be effective also against behaviourally-resistant mosquitoes. As with the eave tubes approach, here too there are new opportunities for resistance management, as different insecticides or insecticide classes can be used. Indeed, earlier studies, also in Tanzania had demonstrated that insecticide treated eave baffles, or baffles treated with spores of entomopathogenic fungi provide can effective control disease-transmitting mosquitoes in households [40] and synthetic odour-baited stations [41].

An important point made in the WSEBs publication is that because of insecticide resistance and logistical challenges, the world has moved towards newer, but inevitably more expensive insecticide formulations, such as the long-lasting formulations of pirimiphos methyl [36]. As a consequence, coverage with IRS has had to be reduced significantly to match budget constraints. It is likely that when new chemical compounds become available for IRS and LLINs, they too will be more expensive, as is already the case with new generation LLINs, such as PermaNet^®^ 3.0, Olyset^®^ Duo and Olyset^®^ Plus, which cost significantly more than the original conventional versions of these net families, i.e. PermaNet^®^ 2.0 and Olyset^®^ nets. Approaches such as eave tubes and the WSEBs, could therefore also enable public health authorities to maintain high coverage of insecticide-based protection, while effectively countering previous operational failures associated with physiological and behavioural resistance, as well as poor user compliance. These advantages are in addition to the magnification of household-level effects to achieve communal effects, by maximizing mortality against disease-transmitting mosquitoes of different species.

## The simplified concept of eave tubes implies scalability

The eave tubes technology, offers a unique pathway to potentially breaking most barriers to translation of research findings into marketable products. It is particularly interesting that developers of this technology chose to refer to it simply as eave tubes, yet the actual approach actually includes filling eave gaps and inserting the tubes in remnant spaces and screening windows. This branding carries a message of scalability, especially since general house improvement is often interpreted as cumbersome, expensive and not easy to implement in large scale. The eave tubes concept could simplify house improvement into a tangible, household product that users can purchase and integrate into their houses during or after construction. It is also foreseeable that commercial companies could manufacture and distribute standardized versions of the products independently of, but complementary to the construction industry, which is growing in many low-income communities but is still non-standardized.

## Eave tubes could achieve high communal effects but combinations with LLINs and IRS would not necessarily be synergistic

The empirical data provided in the series of publications reviewed here was obtained from the laboratory or in semi-field enclosures with a model village having six houses, four of which were fitted with eave tubes [17]. In these tests, the eave tubes approach greatly reduced mosquito house entry, mosquito survival and overall densities. There was however no field data to demonstrate effects on the above parameters, or on epidemiological profiles of mosquito-borne infections. Nevertheless, given the specific properties demonstrated in these initial studies, it is highly likely that the technology could have substantial population-level effects, with limited operational failures associated with physiological resistance, given the use of electrostatic coatings [27].

To illustrate this potential for accruing communal benefits, the team, in their third article in the series described a model of possible transmission control scenarios and outcomes, across different levels of coverage [18]. Here, they distinguished between household-level and community-level impact, focusing on the magnitude of benefits accruable by people living in households without eave tubes. This in silico assessment largely confirmed the expected communal benefits, which surprisingly are accruable even at very low coverage limits [18]. The authors estimated that even if only one-third of dwellings have eave tubes, there could be at least 70% reduction in the relative transmission potential [a derived metric used to show the reduction in infectious mosquito bites in intervention, relative to non-intervention scenarios; and which in several settings can be proportionate to reductions in malaria transmission intensities as measured by entomological inoculation rates (EIRs)]. The rate of change associated with increasing coverage was slow at both low and high intervention coverages, but climbed steeply in the middle ranges of coverage, forming a sigmoidal relationship characteristic of epidemiological effects of many interventions deployed over large communities. As a result, it appears that once coverage with eave tubes reaches about three quarters of the households, further coverage increases may achieve only marginal gains, for which further financial investments may not be necessarily preferable over existing interventions.

The authors also assessed potential benefits of combining eave tubes with LLINs and IRS. While their semi-field tests had demonstrated clear incremental benefits of adding eave tubes onto LLINs [17], results of their model simulations suggest that these incremental benefits are at best additive, rather than synergistic; meaning that combinations of eave tubes and either LLINs or IRS would be desirable only where there are no overlaps, i.e. if they are in different households [18]. Similar to what has been observed where LLINs and IRS are themselves combined, even this additive value of simultaneously using eave tubes and either of these tools will become obvious only where either method is compromised in one way or another [42]. If the eave tubes also use pyrethroids, or similar insecticide class as used in ether LLINs or IRS, then even the marginal gains associated with resistance management would potentially be lost.

There are other important inferences from the design and assumptions of these simulations to be noted. First, other than the oversimplification of the ecosystem as admittedly reported [18], the model also did not consider complexities in nature and instead presents all outcomes on a comparative rather than absolute scale. Also, at least one of the model assumptions, that mosquitoes either feed or die, is an oversimplification, clearly leading to a misrepresentation of overall epidemiological impact. In mosquito life cycle processes, these two events, blood-feeding and dying, are not mutually exclusive, and can occur sequentially, especially in control houses without eave tubes. While mosquitoes would make contact with the eave tube surfaces mostly when they are attempting to enter houses, contacts with LLINs and IRS occur only among mosquitoes that are already inside the houses. Interpretation of the potential effects of these differences technologies should take these differences into account, since implications for individual level protection are also dissimilar. Second, the toxicity component of the eave tube approach is validated as the main source of the communal benefits associated with this technology, as opposed to the deflection associated with the physical barrier effects when the houses are modified. Killeen et al. previously obtained similar findings while assessing whether toxicity or repellency should be prioritized when developing new tools with community-level benefits [43]. Lastly, though the initial studies demonstrated good efficacy of pyrethroids-treated eave tubes against resistant mosquitoes, such approaches could theoretically exacerbate the insecticide pressure on malaria mosquitoes. It may, therefore, be desirable that such an approach is avoided and that alternative insecticide classes used instead.

## Coverage, equity and distribution methods: who should get the eave tubes first, and at what cost?

Other than the biological and epidemiological indicators of success, it is important to also consider optimal strategies for achieving maximum coverage and cost-effectiveness. In line with the 80/20 statistical rule, epidemiologists have observed for years that in a typical community, only a small percentage of people or households (usually estimated as ~20%) carry most of the total burden of infectious diseases (usually estimated as ~80%), and therefore effective interventions intended to achieve mass effect should prioritize these high risk groups effectively [44]. Policy makers and implementers will need to address questions such as which segments of the population should have priority access to the eave tubes technology, and which distribution system, i.e. mass campaigns, subsidized access or private sector retail markets, would be the most appropriate path to providing access. A specific concern regarding eave tubes is that the technology in its current form is not applicable across all house types and therefore not universally applicable. There are many communities and families living in low-grade or rudimentary housing structures, not readily amenable to the eave tubes approach. This requires careful considerations, particularly on whether eave tubes could realistically be used to target the lowest-income, who are often also the most at risk populations.

First, what can be learned from the communal benefits observed even under low level coverage with eave tubes [18]? On one hand, the findings indicate that where eave tubes are delivered through mass distribution approaches or through subsidies prioritizing the lowest income and highest at-risk groups as done with LLINs [45–48], maximum benefits could be achieved, probably tilting the epidemiological transition towards malaria elimination. The main question here is whether such approaches would actually be practical for eave tubes and whether the people most at risk are also the ones whose houses are readily amenable to the technology. Unfortunately, the lowest-income people tend to also be the ones with poorest house designs, which will be least amenable to eave tubes.

If on the other hand the product is provided purely as a retail product, it will still be necessary to create a situation where the lowest socio-economic groups, and highest at risk groups are excluded because they cannot afford, and because their house structures are not readily amenable to the technology. In such cases, the overall communal benefit will be minimal and the residual disease transmission will most likely remain concentrated in the poorest quintiles and high-risk households. This equity problem has been demonstrated previously in the case of insecticide-treated net distribution approaches [49] and used to justify mass distribution targeting low-income groups every few years, complemented by sustenance efforts [46].

Perhaps a mixture of public and private sector approaches is needed where mass distribution prioritizing lowest-income and highest at-risk groups with comprehensive house-improvement programme, is combined with a vibrant private sector system offering the eave tubes for the middle- and high-income families at affordable prices continuously. This will sustain high coverage and maximize epidemiological benefits across entire communities. Similar combined approaches have been demonstrated to work well for LLINs [47] and could be optimized for eave tubes.

These are important considerations that the eave tubes technology champions need to consider in the years ahead. Unfortunately, it is not possible to assess these options directly given that the model used by the team was a simple deterministic representation mosquito life cycle processes with no human demographic component [18]. The model assumed homogeneity in the human populations, in terms of exposure to disease risk and also access to the technology and pre-existing housing designs, which in reality will not be the case.

## Conclusion

Improved housing has the potential to reduce malaria transmission nearly as much as insecticidal bed nets, with potential additional benefits such as control of other mosquito-borne illnesses and overall improvements in wellbeing. The eave tube technology offers an innovative new approach to implementing house improvements, by creating a new scalable product that can be obtained separately and integrated in houses during or after construction. Going forward, it will be important to generate adequate epidemiological and cost-effectiveness evidence to ascertain whether the approach could be considered for large-scale implementation. It will also be important to define the best approaches for implementation including how best to prioritize access based on socio-economic groups as well as level of epidemiological risk. Implementing agencies and decision-makers will also need to consider the key technical limitations such as the need for extensive house modifications before eave tubes are inserted, non-suitability of certain house types for this technology, the potential ineligibility of the poorest households, and poor synergies when used simultaneously with combined with LLINs or IRS in same households. Overall this paradigm significantly improves delivery of insecticides against disease-transmitting mosquitoes and provides major opportunities for scaling-up the long-neglected concept of house improvement as a malaria intervention.

## References

[1] Nájera J, González-Silva M, Alonso PL (2011). Some lessons for the future from the Global Malaria Eradication Programme (1955–1969). PLoS Med..

[2] Cueto M (2004). The origins of primary health care and selective primary health care. Am J Public Health.

[3] WHO. The Abuja declaration and the plan of action. An extract from the African Summit on Roll Back Malaria. Abuja WHO/CDS/RBM/2000; 2000.

[4] WHO (2016). World Malaria Report 2016.

[5] Bhatt S, Weiss D, Cameron E, Bisanzio D, Mappin B, Dalrymple U (2015). The effect of malaria control on *Plasmodium falciparum* in Africa between 2000 and 2015. Nature.

[6] Tusting LS, Thwing J, Sinclair D, Fillinger U, Gimnig J, Bonner KE (2013). Mosquito larval source management for controlling malaria. Cochrane Database Syst Rev.

[7] WHO. Global technical strategy for malaria 2016–2030. World Health Organization; 2015.

[8] The malERA Consultative Group on Vector Control (2011). A research agenda for malaria eradication: vector control. PLoS Med..

[9] Sachs JD (2012). From millennium development goals to sustainable development goals. Lancet.

[10] Tusting LS, Bottomley C, Gibson H, Kleinschmidt I, Tatem AJ, Lindsay SW (2017). Housing improvements and malaria risk in sub-Saharan Africa: a multi-country analysis of survey data. PLoS Med..

[11] Killeen GF, Govella NJ, Lwetoijera DW, Okumu FO (2016). Most outdoor malaria transmission by behaviourally-resistant *Anopheles arabiensis* is mediated by mosquitoes that have previously been inside houses. Malar J..

[12] Ogu VI, Ogbuozobe JE (2001). Housing policy in Nigeria: towards enablement of private housing development. Habitat Int..

[13] Karl G. Housing policy in developing countries. The narodowy bank polski workshop: recent trends in the real estate market and its analysis. 2013. doi:10.2139/ssrn.2842193. Available at SSRN: https://ssrn.com/abstract=2842193

[14] Mubila M, Aissa M-SB, Lufumpa CL. The middle of the pyramid: dynamics of the middle class in Africa. African Development Bank, Market Brief, 20 April 2011.

[15] Ogoma SB, Kannady K, Sikulu M, Chaki PP, Govella NJ, Mukabana WR (2009). Window screening, ceilings and closed eaves as sustainable ways to control malaria in Dar es Salaam, Tanzania. Malar J..

[16] Knols BG, Farenhorst M, Andriessen R, Snetselaar J, Suer RA, Osinga AJ (2016). Eave tubes for malaria control in Africa: an introduction. Malar J..

[17] Sternberg ED, Ng’habi KR, Lyimo IN, Kessy ST, Farenhorst M, Thomas MB (2016). Eave tubes for malaria control in Africa: initial development and semi-field evaluations in Tanzania. Malar J..

[18] Waite JL, Lynch PA, Thomas MB (2016). Eave tubes for malaria control in Africa: a modelling assessment of potential impact on transmission. Malar J..

[19] Lindsay SW, Snow RW (1988). The trouble with eaves: house entry by vectors of malaria. Trans R Soc Trop Med Hyg.

[20] Okumu FO, Moore J, Mbeyela E, Sherlock M, Sangusangu R, Ligamba G (2012). A modified experimental hut design for studying responses of disease-transmitting mosquitoes to indoor interventions: the Ifakara experimental huts. PLoS ONE.

[21] Lindsay S, Jawara M, Paine K, Pinder M, Walraven G, Emerson P (2003). Changes in house design reduce exposure to malaria mosquitoes. Trop Med Int Health..

[22] Kirby MJ, Ameh D, Bottomley C, Green C, Jawara M, Milligan PJ (2009). Effect of two different house screening interventions on exposure to malaria vectors and on anaemia in children in The Gambia: a randomised controlled trial. Lancet.

[23] Spitzen J, Koelewijn T, Mukabana WR, Takken W (2016). Visualization of house-entry behaviour of malaria mosquitoes. Malar J..

[24] Knols B, Bart GJ (2014). Six new ways to control malaria mosquitoes. Knols’ Blog.

[25] Maurice J (2015). UN set to change the world with new development goals. Lancet.

[26] Osinga A, Suer R, Farenhost M, Knols B, Thomas M, Heinig R, et al. A complex of structures for delivering pesticidal agents to arthropods. In: European Patent Register (Office EP ed., vol. EP2859794B1, A01M29/12, A01M1/20, A01M29/34, A01N63/04 edition). For All designated States: In2Care Holding BV; 2015.

[27] Andriessen R, Snetselaar J, Suer RA, Osinga AJ, Deschietere J, Lyimo IN (2015). Electrostatic coating enhances bioavailability of insecticides and breaks pyrethroid resistance in mosquitoes. Proc Nat Acad Sci USA.

[28] Putro MG. Electrostatic air filter device. Google Patents; 1998.

[29] Reiss-Schmidt T. Pollen or insect screen for applying to openings in buildings such as windows, doors or similar. Google Patents; 2002.

[30] Santhouse D. Hairdryer with electrostatic precipitator and filter cleanout warning. Google Patents; 2007.

[31] Latheef MA, Carlton JB, Kirk IW, Hoffmann WC (2009). Aerial electrostatic-charged sprays for deposition and efficacy against sweet potato whitefly (*Bemisia tabaci*) on cotton. Pest Manag Sci.

[32] Huang J, Stelinski L, Gut L (2010). Mating behaviors of *Cydia pomonella* (Lepidoptera: Tortricidae) as influenced by sex pheromone in electrostatic powder. J Econ Entomol.

[33] WHO. Test procedures for insecticide resistance monitoring in malaria vector mosquitoes. Geneva: World Health Organization; 2013.

[34] WHO Pesticide Evaluation Scheme (WHOPES). http://www.who.int/whopes/en/. 10.1016/0169-4758(88)90082-8 15463084

[35] Okumu FO, Chipwaza B, Madumla EP, Mbeyela E, Lingamba G, Moore J (2012). Implications of bio-efficacy and persistence of insecticides when indoor residual spraying and long-lasting insecticide nets are combined for malaria prevention. Malar J..

[36] Oxborough RM, Kitau J, Jones R, Feston E, Matowo J, Mosha FW (2014). Long-lasting control of *Anopheles arabiensis* by a single spray application of micro-encapsulated pirimiphos-methyl (Actellic^®^ 300 CS). Malar J..

[37] Killeen G, Masalu JP, Chinula D, Fotakis EA, Kavishe D, Malone D (2017). Control of malaria vector mosquitoes by insecticide-treated combinations of window screens and eave baffles. Emerg Infect Dis.

[38] Smith A (1965). A verandah-trap hut for studying the house-frequenting habits of mosquitos and for assessing insecticides. I.—a description of the verandahtrap hut and of the studies on the egress of *Anopheles gambiae* Giles and *Mansonia uniformis* (Theo.) from an untreated hut. Bull Entomol Res.

[39] Smith A, Hudson JE (1972). A modification to an experimental hut to reduce mosquito eaves egress.

[40] Mnyone LL, Lyimo IN, Lwetoijera DW, Mpingwa MW, Nchimbi N, Hancock PA (2012). Exploiting the behaviour of wild malaria vectors to achieve high infection with fungal biocontrol agents. Malar J..

[41] Lwetoijera DW, Sumaye RD, Madumla EP, Kavishe DR, Mnyone LL, Russell TL (2010). An extra-domiciliary method of delivering entomopathogenic fungus, *Metharizium anisopliae* IP 46 for controlling adult populations of the malaria vector, *Anopheles arabiensis*. Parasites Vectors..

[42] Lines J, Kleinschmidt I (2015). Is malaria control better with both treated nets and spraying?. Lancet.

[43] Killeen GF, Okumu FO, N’guessan R, Coosemans M, Adeogun A, Awolola S (2011). The importance of considering community-level effects when selecting insecticidal malaria vector products. Parasites Vectors..

[44] Woolhouse MEJ, Dye C, Etard JF, Smith T, Charlwood JD, Garnett GP (1997). Heterogeneities in the transmission of infectious agents: implications for the design of control programs. Proc Nat Acad Sci USA.

[45] Killeen GF, Tami A, Kihonda J, Okumu FO, Kotas ME, Grundmann H (2007). Cost-sharing strategies combining targeted public subsidies with private-sector delivery achieve high bednet coverage and reduced malaria transmission in Kilombero Valley, southern Tanzania. BMC Infect Dis.

[46] Teklehaimanot A, Sachs JD, Curtis C (2007). Malaria control needs mass distribution of insecticidal bednets. Lancet.

[47] Khatib RA, Killeen GF, Abdulla SM, Kahigwa E, McElroy PD, Gerrets RP (2008). Markets, voucher subsidies and free nets combine to achieve high bed net coverage in rural Tanzania. Malar J..

[48] Bonner K, Mwita A, McElroy PD, Omari S, Mzava A, Lengeler C (2011). Design, implementation and evaluation of a national campaign to distribute nine million free LLINs to children under five years of age in Tanzania. Malar J..

[49] Noor AM, Amin AA, Akhwale WS, Snow RW (2007). Increasing coverage and decreasing inequity in insecticide-treated bed net use among rural Kenyan children. PLoS Med..

